# Genome Sequence of *Bibersteinia trehalosi* Strain Y31 Isolated from the Pneumonic Lung of a Bighorn Sheep

**DOI:** 10.1128/genomeA.00722-16

**Published:** 2016-07-21

**Authors:** Abirami Kugadas, Jodi L. Humann, Sebastián Aguilar Pierlé, Subramaniam Srikumaran, Kelly A. Brayton

**Affiliations:** a Program in Genomics, Department of Veterinary Microbiology and Pathology, Washington State University, Pullman, Washington, USA; b Department of Horticulture, Washington State University, Pullman, Washington, USA

## Abstract

Here, we report the genome sequence for *Bibersteinia trehalosi* strain Y31, isolated from the lungs of a bighorn sheep (*Ovis canadensis*) that had succumbed to pneumonia, which exhibits proximity-dependent inhibition (PDI) of *Mannheimia haemolytica*. The sequence will be used to understand the mechanism of PDI for these organisms.

## GENOME ANNOUNCEMENT

The Gammaproteobacterial species *Pasteurella haemolytica* was subdivided into biotypes A and T based on the ability to ferment either arabinose or trehalose. In 1990, the T biotypes were reclassified and named *Pasteurella trehalosi* (1). In a recent reorganization, *P. trehalosi* was once again reclassified and given the name *Bibersteinia trehalosi*, in honor of Ernst Biberstein, who did much of the characterization work on the organism (2). *Bibersteinia trehalosi* is a Gram-negative, nonmotile, and rod-shaped or pleomorphic bacterium that is associated with pneumonia in bighorn sheep (BHS) (3). *Mannheimia haemolytica* strains that produce leukotoxin consistently cause fatal pneumonia in BHS under experimental conditions, but surprisingly, *M. haemolytica* is isolated less frequently than *B. trehalosi* from pneumonic lung tissue. We previously reported that a broad range of *B. trehalosi* strains are able to inhibit the growth of *M. haemolytica* in a proximity-dependent inhibition (PDI) manner, and this inhibition contributes to the infrequent detection of *M. haemolytica* from pneumonic lungs (4, 5). To identify the genomic component that provides this inhibitory effect, we selected an inhibitory strain of *B. trehalosi*, Y31, isolated from the pneumonic lung of a BHS (4), for complete genome sequencing.

*B. trehalosi* Y31 was grown in brain heart infusion medium (Remel, Lenexa, KS), and genomic DNA was isolated using the QIAamp DNA minikit (Qiagen, Valencia, CA). The genome was sequenced on a PacBio RS instrument (Pacific Biosciences, Menlo Park, CA) using single-molecule real-time (SMRT) sequencing technology (6), yielding 86× coverage. The sequences from 12 SMRT cells were assembled with SMRT Pipe version 2.0 (Pacific Biosciences) using the HGAP protocol. The assembly yielded 17 contigs, with an *N*_50_ length of 351,951 bases. Optical mapping (OpGen, Gaithersburg, MD) was used to order the contigs, and gap-spanning PCR was used to link the contigs. Finally, two contigs were produced that could not be definitively connected; however, optical mapping suggests the gaps are 2,872 and 31,034 bp. The genome sequence includes 2,334,734 bp. The G+C content was 41%. The genome sequence was subjected to autoannotation at NCBI using the Prokaryotic Genome Annotation Pipeline (7) and yielded 2,240 genes, 5 rRNA operons, 56 tRNA genes, and one noncoding RNA (ncRNA).

PDI is often effected by a bacteriocin that may be encoded on a plasmid or on the chromosome (8). This strain contained no plasmids; thus, the gene(s) responsible for PDI is chromosomally located. In addition to the effector molecule, a transporter protein would be necessary to secrete/transport the effector to the target cell. We examined the genome for transporters and detected 274, with most belonging to the ABC superfamily of transport proteins.

### Nucleotide sequence accession number.

The genome sequence has been deposited at GenBank under accession no. JACI00000000**.**
